# A 10-Year Probability Deep Neural Network Prediction Model for Lung Cancer

**DOI:** 10.3390/cancers13040928

**Published:** 2021-02-23

**Authors:** Hsiu-An Lee, Louis R. Chao, Chien-Yeh Hsu

**Affiliations:** 1 Department of Computer Science and Information Engineering, Tamkang University, New Taipei 251, Taiwan; billy72325@gmail.com (H.-A.L.); chaory@gmail.com (L.R.C.); 2 National Health Research Institutes, Zhunan 350, Taiwan; 3 Department of Information Management, National Taipei University of Nursing and Health Sciences, Taipei 112303, Taiwan; 4 Master Program in Global Health and Development, Taipei Medical University, Taipei 110, Taiwan

**Keywords:** lung cancer, prediction model, early diagnosis, health prevention, machine learning, deep neural network model

## Abstract

**Simple Summary:**

Cancer is the leading cause of death in Taiwan. Compared with other types of cancer, the incidence of lung cancer is high. In this study, the National Health In-surance Research Database (NHIRDB) was used to determine the diseases and symptoms associ-ated with lung cancer, and a 10-year probability deep neural network prediction model for lung cancer was developed. The proposed model could allow patients with a high risk of lung cancer to receive an earlier diagnosis and support the physicians’ clinical decision-making. As a result, a total of 13 diseases were selected as the predicting factors. The proposed model showed an accuracy of 85.4%, a sensitivity of 72.4% and a specificity of 85%, as well as an 87.4% area under ROC (95%, 0.8604–0.8885) model precision. Based on data analysis and deep learning, our prediction model discovered some features that had not been previously identified by clinical knowledge. This study tracks a decade of clinical diagnostic records to identify possible symptoms and comorbidities of lung cancer, allows early prediction of the disease, and assists more patients with early diagnosis.

**Abstract:**

Cancer is the leading cause of death in Taiwan. According to the Cancer Registration Report of Taiwan’s Ministry of Health and Welfare, a total of 13,488 people suffered from lung cancer in 2016, making it the second-most common cancer and the leading cancer in men. Compared with other types of cancer, the incidence of lung cancer is high. In this study, the National Health Insurance Research Database (NHIRDB) was used to determine the diseases and symptoms associated with lung cancer, and a 10-year probability deep neural network prediction model for lung cancer was developed. The proposed model could allow patients with a high risk of lung cancer to receive an earlier diagnosis and support the physicians’ clinical decision-making. The study was designed as a cohort study. The subjects were patients who were diagnosed with lung cancer between 2000 and 2009, and the patients’ disease histories were back-tracked for a period, extending to ten years before the diagnosis of lung cancer. As a result, a total of 13 diseases were selected as the predicting factors. A nine layers deep neural network model was created to predict the probability of lung cancer, depending on the different pre-diagnosed diseases, and to benefit the earlier detection of lung cancer in potential patients. The model is trained 1000 times, the batch size is set to 100, the SGD (Stochastic gradient descent) optimizer is used, the learning rate is set to 0.1, and the momentum is set to 0.1. The proposed model showed an accuracy of 85.4%, a sensitivity of 72.4% and a specificity of 85%, as well as an 87.4% area under ROC (AUROC) (95%, 0.8604–0.8885) model precision. Based on data analysis and deep learning, our prediction model discovered some features that had not been previously identified by clinical knowledge. This study tracks a decade of clinical diagnostic records to identify possible symptoms and comorbidities of lung cancer, allows early prediction of the disease, and assists more patients with early diagnosis.

## 1. Introduction

Cancer is the leading cause of death in Taiwan. According to the Cancer Registration Report of Taiwan’s Ministry of Health and Welfare, a total of 13,488 people suffered from lung cancer in 2016, making it the second-most common cancer and the most common cancer in men.

Cancer is usually curable by surgery and adjunctive therapy when it is diagnosed in the early stages [1]. An early diagnosis is important in the elderly, even if the patient has other diseases. Surgery can improve a patient’s quality of life, even if the goal is not to extend the life of the patient [2]. The radiation and medical treatment provided after surgery can reduce the risk of the cancer spreading, but these adjuvant treatments can cause temporary harm. For patients over 65 years of age, chemotherapy should be avoided, and if radiation therapy is adopted smaller dosages than usual should be used [3]. Recent clinical research has shown that patients no longer have an upper age limit, but that they are selected according to their general condition (excluding other diseases). Cancer treatments, such as chemotherapy and radiation therapy, often cause more damage and are more severe in older people than in younger people. However, if the patient has no other diseases, a treatment plan can sometimes be prepared in the same way as for a young patient. Regardless of their age, both drug therapy and radiation therapy may have the same effect. Other diseases of the elderly, such as diabetes, vascular disease, and impaired kidney function, may increase the risk of infection, anemia, nausea, depression, and exhaustion [4]. Elderly patients tend to recover more slowly from treatment. 

Therefore, early diagnosis has been receiving increased attention, and more and more research is focusing on disease prediction and detection. Through artificial intelligence calculations and programming, disease prediction models that are based on big data can be constructed. 

## 2. Literature Review

Previous research has developed a prediction model for lung cancer. Cassidy et al. [5] employed the LLP(Liverpool Lung Project) risk model to estimate the probability of lung cancer development with a specific combination of risk factors within a five-year period. These risk factors include the age and sex of the patient, previous malignant tumors, smoking duration, the age of onset of lung cancer, asbestos exposure, cases of lung cancer, and pneumonia history. The area under the Receiver Operating Characteristic curve (ROC) of the model was 0.71. Bach et al. [6] built two models, namely, one for the one-year risk of developing an incidence of lung cancer, and one for computing the risk of dying from lung cancer without a positive diagnosis. The prediction factors of both models include the age and sex of the patient, smoking duration, smoking intensity, the length of time since quitting smoking, and asbestos exposure. The model was built using the Cox proportional hazard regression, and the area under the ROC of the prediction model was 0.72. Many different target disease models have been established by different algorithms, such as the Artificial Neural Network (ANN), Cox regression, logistic regression, and the Support Vector Machine (SVM), for other diseases [7,8,9,10,11]. The results of past related studies, including the number of factors, factors and model performance, and the comparison table is shown in Table 1.

This study developed a 10-year lung cancer risk prediction model based on the Taiwan National Health Insurance Research Database (NHIRD). Potential diseases related to lung cancer were used as the predictive risk factors and were identified by using big data analysis methods, and the prediction model for lung cancer was established by using the Deep Neural Network (DNN) method. From an objective perspective, the model could be used in conjunction with the patients’ health management as a reference for early lung cancer prediction. Patients could also use this model for early screening, based on the risk factors that may be related to lung cancer and, if the results showed an increased possibility of the risk of developing lung cancer, earlier attention could be applied. The model is expected to reduce the risk of lung cancer for patients with advanced related diagnoses through early screening, and furthermore, to reduce the burden on medical services.

## 3. Method

### 3.1. Data Resource and Processing

This study sourced the data from the NHIRD, which dataset covers more than 99% of the national health insurance data of the Taiwanese population [12], including their medical records, diagnosis records, medication records, surgical records, treatment records, and other detailed clinical information. The dataset that was used in this study is published by the NHI in Taiwan and contains all the above-mentioned information on 2,000,000 randomly-sampled individuals from the NHIRD. There is no significant difference between the subset samples and the original NHIRD samples regarding the distribution of gender and age. 

Patients who developed lung cancer between 1 January 2000 and 31 December 2009 were eligible for inclusion in this study, and all outpatient records before diagnosis were tracked in order to identify the potential impacts or comorbidities of the disease, and to assist patients with early screening. The target population was lung cancer patients. We identified patients with lung cancer as case groups between 2000–2009, tracked their past diagnostic records, and excluded individuals with other cancer histories. The quadruple number of subjects as control groups were identified by their gender and age distribution [13].

The patients who were recorded in the Cancer Registration Form, and who had a diagnosis code of ICD-O-3, including C340, C341, C342, C343, C348, C349, were identified as the case group, and patients with any other cancer diagnosis were excluded, to make sure that the participants were not undergoing cancer metastasis. The control group was selected by their distribution of gender and age, based on the quadruple number of the case group. Each patient record included the visit date, the patient’s de-identified pseudo ID, gender, age, and diagnosis. All records with errors, such as miscoded diagnoses, typos, or missing data, were excluded.

For clinical characteristics, we screened the patients’ disease history from the NHIRD. The ICD-9-CM (International Classification of Disease-9-Clinical Modification) code was used as the diagnosis code in the NHIRD, and it included the category number (three codes) and the sub-category number (two codes). To avoid including too many disease sub-categories, only the category numbers were used in this study.

**Figure 1:**
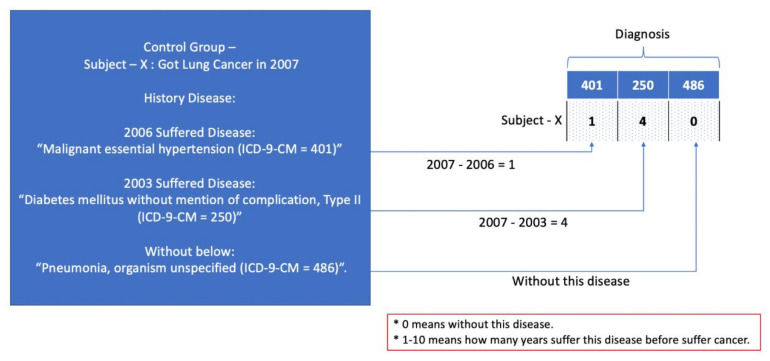
Data processing example.

For the data coding process, the data were transferred, depending on the time lapse between the other diseases and the diagnosis of lung cancer. For example, for a subject who was diagnosed with lung cancer in 2007, to code the history disease before the diagnosis, the data were coded from 0–10, where 0 means that the person was without this disease and 1–10 denotes how many years the person has suffered from this disease before suffering from cancer (the control group subject will be based on mapping the subject to the case group). In this example, the data would be coded as 401 = 1, 250 = 4, and 486 = 0. An example is shown in Figure 1.

### 3.2. Research Process

**Figure 2:**
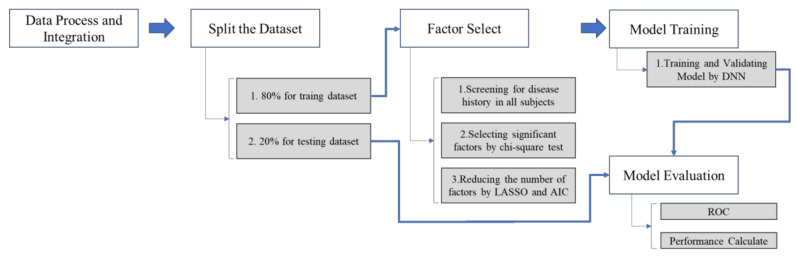
Research process.

Figure 2 shows the research process. Based on NHIRD, the outpatient records of both the case group and the control group were reviewed retrospectively. After data processing and integration, the research process followed the next three steps. 

Step 1. The full dataset was separated into training (80%) and testing (20%) datasets.

Step 2. Select significant factors (predictors) by using the Least Absolute Shrinkage and Selection Operator (LASSO) and Akaike Information Criterion (AIC) from the whole dataset.

Step 3. The model was built using the training dataset by DNN.

Step 4. The model was evaluated by the testing dataset.

Step 5. The performance of the model is presented by accuracy, AUROC, sensitivity, and specificity.

### 3.3. Factor Selection

For the selection of the influencing factors, all previous disease data were included, and the case group was compared with the control group to find the significant correlation factors. During the comparison process, the factors that had been confirmed by the literature review were also included such as asthma, chronic obstructive pulmonary disease, tuberculosis, emphysema, chronic bronchitis, silicosis, lung trauma, fibrosis, and pleural thickening. The number of selection factors was reduced before finding the best factors for the design of the prediction model.

The factors were reduced by adopting stepwise regression and by using the AIC and the LASSO. LASSO is a regression analysis method that performs both feature selection and regularization, in order to enhance a model’s predictive accuracy and interpretability. It forces the sum of the absolute values of the regression coefficients to be less than a fixed value (e.g., forcing some regression coefficients to become 0), which results in effectively selecting a simpler model that unites the covariates that correspond to these regression coefficients. This method is similar to ridge regression, in which the sum of the squares of the regression coefficients is forced to be less than a certain value. The difference is that ridge regression only changes the value of the coefficient, without setting any values to zero.

LASSO is designed by the least squares method and assumes that a sample includes N events, with each event consisting of p covariates and an output value of *y*. Let *y_i_* be the output value of the *i*th case, and $x_{i}={\left(x_{1},\;x_{2},\;x_{3},\;\ldots ,\;x_{p}\right)}^{T}$ the covariate vector of the *i*th case; the target equation to be calculated by LASSO is:

$$\underset{\beta_{o},\;\beta }{\min}\left\{\frac{1}{N}\overset{N}{\underset{i=1}{\sum }}{\left(y_{i}-\beta_{0}-x_{i}^{T}\beta \right)}^{2}\right\}\;subject\;to\;\overset{p}{\underset{j=1}{\sum }}\left|\beta_{j}\right|\leq t$$

where *t* is a pre-specified free parameter that determines the amount of regularization. Suppose $t_{0}$ = $\sum \left|{\hat{B}}_{j}^{OLS}\right|$; when $t>t_{0}$, ${\hat{B}}_{j}^{OLS}$ means ${\hat{B}}_{j}$ will be calculated by an ordinary least squares’ statistical analysis, and the parameter coefficient estimator of the LASSO regression will be equivalent to the least squares difference parameter coefficient estimator. When $t\leq t_{0}$, part of the parameter coefficient of the LASSO regression will be reduced to zero, thus completing the dimensionality reduction (feature extraction).

### 3.4. Model Training

After the factor selection, the prediction model was designed by using an ANN. An ANN simulates a biological neural network by using information systems and hardware [14]. The neural network is composed of many artificial neurons, which can be divided into the feedforward neural network and the recurrent neural network, with the feedforward type being the most widely used.

The feedforward neural network includes input nodes, as well as neurons, in the hidden layer, and it results in the output layer. In general, the multiple hidden layers, and neurons in the same layer are not connected to each other, but are rather connected to neurons in different layers, and signal transmissions occur in one direction only. 

**Figure 3:**
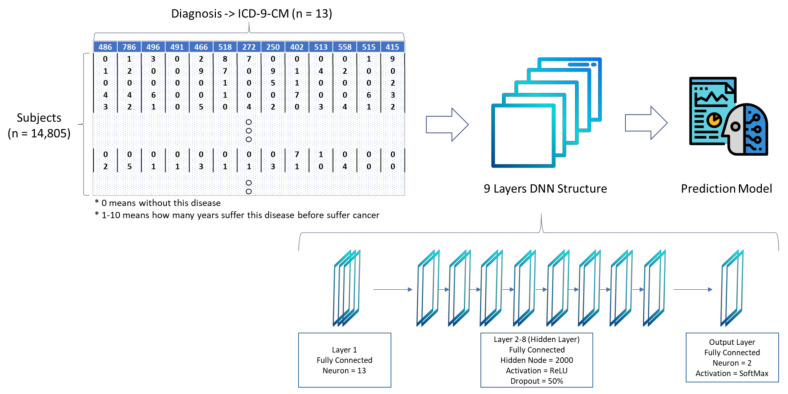
Deep Neural Network (DNN) model structure.

In brief, output *Y_i_* can be calculated as $Y_{j}=\overset{n}{\underset{k=1}{\sum }}\overset{m}{\underset{i=1}{\sum }}H_{ijk}X_{i}$, where Σ is the summation and *H_ijk_* is the neuron value to be learned. Back propagation is most representative in the feedforward learning method, which first calculates the output error term and then feeds the output error term from the output layer to the hidden layer through the neural link. Backpropagation calculates the loss function relative to the network weight gradient for a single input-output value. Through repeated iterations, the neural network is trained to make the output value close to the actual value. In this study, a nine-layer deep ANN was used for model training. Each layer included 2000 neurons, and the ReLu (Rectified Linear Unit) activation function was used. To avoid model over fitting, the dropout layer, which was a dropout of 50% neurons, was used randomly between Layers One and Two. The model structure is shown in Figure 3.

### 3.5. Model Evaluation

The Area Under the Receiver Operating Characteristic curve (AUROC) was used to evaluate the performance of the model in this study. When the area is large, it means that the prediction of the model is more significant. In other words, when AUROC is close to 1, the prediction precision of the diagnosis is higher, and when it is close to 0.5, the precision rate of the model is lower [15]. 

A classification model (classifier or diagnosis) is the mapping of instances between certain groups. The classifier or diagnosis result can be a real value (classifier by a threshold) or it can be a discrete class label, indicating one of the classes.

This study designed a two-class prediction problem (binary classification), in which the results were labeled either as positive (p) or negative (n). There were four possible results from the simple problem: 

1. True Positive (TP), in which the results from a prediction are positive and the actual value is also positive.

2. False Positive (FP), in which the results from a prediction are positive and the actual value is negative.

3. True Negative (TN), in which the results from a prediction are negative and the actual value is also negative.

4. False Negative (FN), in which the results from a prediction are negative and the actual value is positive.

Based on the False Positive Rate (FPR) as the X axis and the True Positive Rate (TPR) as the Y axis, the ROC curve can be drawn on the coordinate plane.

Given a binary classification model and its threshold, a (X = FPR, Y = TPR) coordinate point can be calculated from the (positive/negative) true and predicted values of all samples.

The diagonal from (0, 0) to (1,1) divides the ROC space into the upper left/lower right areas. The points above this line represent a good classification result (better than random classification), while the points below this line represent a poor classification result (inferior to random classification).

The perfect prediction point is a point in the upper left corner. At the ROC space coordinate (0,1) point, X = 0 means no false positive and Y = 1 means no false negative (all positives are true positive); that is, regardless of whether the classifier output is positive or negative, it is 100% correct. A random prediction will result in a point on the diagonal from (0, 0) to (1, 1) (also called the no-recognition rate line).

When judging the quality of the model, in addition to the AUROC graph, the discriminating power of the ROC can also be determined. The AUC curve ranges from 0 to 1, and a larger value is preferred. The following are the general discriminant rules for AUC values:

AUC = 0.5 (no discrimination or without discrimination)

0.7 ≦ AUC ≦ 0.8 (acceptable discrimination)

0.8 ≦ AUC ≦ 0.9 (excellent discrimination)

0.9 ≦ AUC ≦ 1.0 (outstanding discrimination)

### 3.6. Tool

The data statistic and model structure were completed by the R program. The LASSO statistics were completed by applying the Glmnet package, and the ANN model was constructed by using the MXNet package. Finally, the ROC curve analysis was accomplished by using the Plotly package (Plotly, CA, USA). 

## 4. Results

### 4.1. Demography

This study identified 3448 subjects who received a lung cancer diagnosis from 1 December 2000 to 30 December 2009. A total of 132 patients were excluded due to typos or missing data. Finally, a total of 3316 subjects were included in the case group for this study. A total of 13,264 subjects were selected by their gender and age distribution as the control group, of which 1775 subjects were excluded, due to typos or missing data. 

The demographic and clinical characteristics of the 3316 subjects in the case group and the 11,489 subjects in the control groups are summarized in Table 2. In order to prevent the model from being affected by age and gender factors, the control group data screening was completed, based on the gender and age distribution. The gender and age distribution of the two groups were similar. The average age of case group subjects (patients with cancer) was 68.36 ± 12.3 years, while the average age of the control group (no cancer) was 68.08 ± 12.3 years. Males made up 67% of the subjects in both groups. 

As can be seen from the basic data, 66% of the subjects were male and 34% of the subjects were female. Regarding the age distribution, 75% of the patients were elderly (60–109 years old).

The distribution of clinical characteristics of the case group are as follows: 12% of the subjects had pneumonia, organism unspecified; 41% of the subjects had respiratory abnormalities, unspecified; 17% of the subjects had NEC (Necrotizing enterocolitis) chronic airway obstruction, not elsewhere classified; 27% of the subjects had simple chronic bronchitis; 55% of the subjects had acute bronchitis; 10% of the subjects had pure hypercholesterolemia; 18% of the subjects had diabetes mellitus without mention of complication, Type II; 20% of the subjects had malignant hypertensive heart disease without congestive heart failure; and 28% of the subjects had other unspecified non-infectious gastroenteritis and colitis. 

The distribution of the clinical characteristics of the control group are as follows: 5% of the subjects had pneumonia, organism unspecified; 31% of the subjects had respiratory abnormalities, unspecified; 11% of the subjects had NEC chronic airway obstruction, not elsewhere classified; 21% of the subjects had simple chronic bronchitis; 51% of the subjects had acute bronchitis; 14% of the subjects had pure hypercholesterolemia; 24% of the subjects had diabetes mellitus, without mention of complications, Type II; 26% of the subjects had malignant hypertensive heart disease, without congestive heart failure; and 34% of the subjects had other unspecified non-infectious gastroenteritis and colitis.

The distribution of clinical characteristics indicated that the proportion of lung disease in the case group was higher than that in the control group; however, chronic diseases, such as diabetes and hypertension, were lower in the case group than in the control group.

### 4.2. Factor Selection

In the case group, a total of 919 disease cases were diagnosed before the diagnosis of lung cancer. The chi-square test was performed on the 919 disease cases, and 132 independent factors were identified as being significantly associated with lung cancer.

The dataset was randomly divided into 80% for the training dataset and 20% for the external validation dataset, based on the same stratified sample size of 4:1 (Control Group: Case Group). 

Finally, 13 factors were selected by executing the LASSO and the Akaike Information Criterion (AIC) from the training dataset. The coefficient and p value of each factor calculated by LASSO and AIC are shown in Table 3. The Akaike information standard was developed by Japanese statistician Hirotugu Akaike [16]. It now forms the basis of the basic statistical paradigm and is also widely used for statistical inference. The Akaike Information Criterion (AIC) is an estimate of the out-of-sample prediction error, and therefore the relative quality of the statistical model for a given data set. Given the set of models used for the data, AIC estimates the quality of each model relative to every other model. Therefore, AIC provides a method of model selection. which can estimate the relative amount of information lost by a given model: the less information the model loses, the higher the quality of the model. When estimating the amount of information lost by the model, AIC will weigh the model’s goodness of fit and model simplicity. In other words, AIC deals with the risk of overfitting and the risk of underfitting. 

In this study, AIC was employed to select the critical factor for establishing the model. Using AIC’s repeated loss of information and the characteristics of the training model, the factors identified by this process were selected as the main factors for final prediction model analysis.

### 4.3. Model Establishment and Evaluation

The dataset was randomly divided into 80% for the training dataset and 20% for the external validation dataset, based on the same stratified sample size of 4:1 (Control Group: Case Group).

The training data is used to train the predictive model. The prediction model was established by the nine layers of the DNN model. The input layer included 13 factors, and each factor was given a rating of between 0–10 (in which 0 indicated no disease and 1–10 indicated how many years a person suffered from the disease before suffering from cancer). Each control group subject can be mapped to the case group subject by their gender and age, and the tracking date of control group subjects is the same as that of the case group.

Usually, the DNN architecture consists of many hidden layers in the network, connected to each other. Under normal circumstances, the structure of the best model can only be determined by training with incremental testing. Different data needs to be used with different number of DNN layer structures to find a better performance model. In this study a total of nine layers of DNN structure including one input layer, seven hidden layers, and one output layer is established. The testing result of different DNN structures is shown in Table 4, in which the prediction model established by the structure has the best performance and is better than other previous studies.

The model is trained 1000 times, the batch size is set to 100, the SGD optimizer is used, the learning rate is set to 0.1, and the momentum is set to 0.1. The parameters of the DNN structure are shown in Table 5.

**Figure 4:**
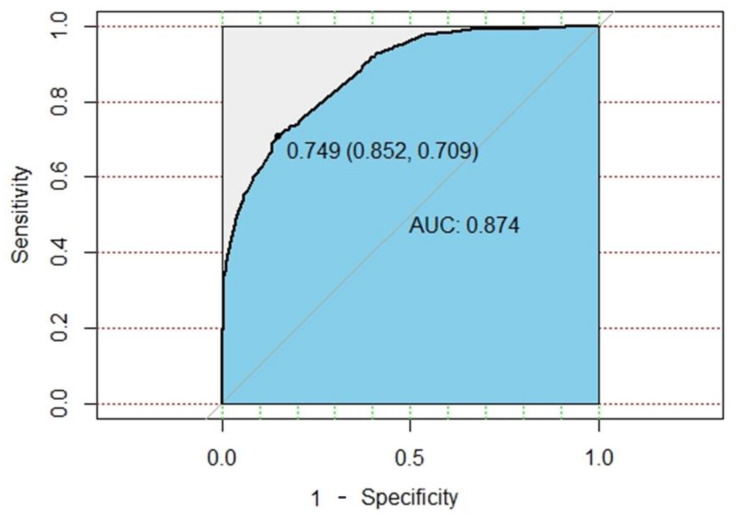
ROC plot of DNN model.

After the model is established, the external validation data is used for external verification of the model. The best threshold of the lung cancer prediction model was 0.749 (95% CI, 0.852 – 0.709), and the performance of the model could attain an accuracy of 85.4% and an AUROC of 0.874 (95% CI, 0.8604–0.8885), with a sensitivity of 72.4% and a specificity of 85%. The ROC plot of the DNN model is shown in Figure 4. 

**Figure 5:**
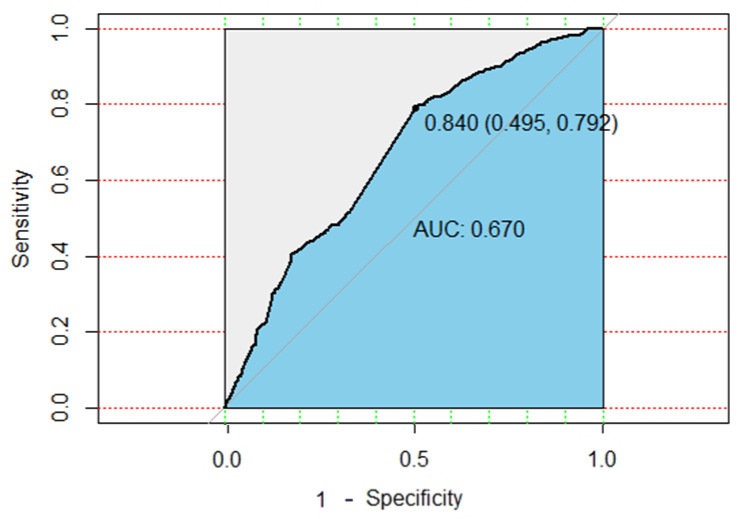
ROC plot of XGBoost model.

This study also uses traditional machine learning methods to compare baseline performance. We use XGBoost for model development and comparison. When training 3000 times, XGBoost model showed an accuracy of 75%, a sensitivity of 18% and a specificity of 91.5%, as well as a 67.3% area under ROC (AUROC) model precision. The ROC plot of XGBOOST model is shown in Figure 5. However, the sensitivity of the XGBoost model is quite low. This study aims to establish an early preventive screening model to assist in personal health management and lung cancer prediction. In this study, sensitivity is the key point of model performance evaluation. Therefore, the performance of the DNN model is better than that of traditional machine learning.

## 5. Discussion and Conclusions

The amount of healthcare data is increasing constantly. Through machine learning technology, large amounts of medical data can be analyzed quickly [17]. Therefore, it is possible to implement machine learning and deep learning models for personalized care relating to clinical decision support and health management. By using an appropriate deep learning ANN prediction model, doctors can make clinical decisions by extracting the minimum amount of necessary data [18]. The model proposed in this study could assist in the early diagnosis of lung cancer, thereby helping to improve the efficiency and quality of clinical diagnoses. The early diagnosis of any disease is essential, as the time of diagnosis is one of the strongest factors in the success rate of any treatment plan. Therefore, the time of diagnosis for each disease was used in our model. Stepwise regression of LASSO and AIC was used cautiously as a feature selection strategy. Data from the NHIRD were used to represent the Taiwanese population, and the predictive model could be integrated easily into the Electronic Medical Record to identify the risk of lung cancer.

This research demonstrated that a neural network model could be used to design a model that recognizes patients who are at risk of lung cancer, especially for those with specific diseases. The model could help physicians to achieve an effective early diagnosis and to minimize potential harm to patients. A number of studies have suggested that several comorbidities are prevalent in patients with lung cancer, including smoking, age [19,20], heavy drinking [20], pneumothorax, COPD, tuberculosis, and hypertension [21,22,23,24]. Although those studies have shown encouraging results, they have some limitations, as most of them need questionnaire answers to calculate the risk, which may not be available in all clinical and health management environments.

In this study, 13 diseases (factors) were selected, not only by using statistical algorithms, but also by confirming the clinical evidence [21,22,23,24]. The 13 diseases included lung-, diabetes-, hypertension-, and heart-related diseases, which are closely-related to lung cancer. These 13 diseases were used to calculate the risk of lung cancer. In contrast to previous studies, questionnaires on smoking and tuberculosis were not necessary in the proposed model, thus making it beneficial for rapid clinical screening, as the patients’ personal health records or electronic health records could be used for rapid screening without the need for questionnaires. The developed models could be used for personal health management.

Previous research models for lung cancer prediction have produced various AUROC results. The Bach model, proposed by Bach et al., has a 0.72 AUROC [6], the Liverpool lung project model, proposed by Cassidy et al., has a 0.71 AUROC [5], the Spitz model, proposed by Spitz et al., has a 0.57–0.63 AUROC [8], the African-American model, proposed by Etzel et al., has a 0.75 AUROC [10], the PLCO_M2012_ model, proposed by Tammemagi et al., has a 0.803 AUROC [7], and the Hoggart model, proposed by Hoggart et al., has a 0.843 AUROC. The results indicated that the proposed model had a higher AUROC. Although the model had a high performance, differences in data resources, datasets, and features could cause differences in the predictive performance. 

Based on the data and deep learning analyses, this study identified some features that could be explained by clinical knowledge. For example, there was no evidence that other unspecified non-infectious gastroenteritis and colitis were directly related to lung cancer; however, it had a high weighting factor in our model. Eliminating this factor from the DNN model training would cause the performance of the model to be greatly reduced.

Future work could attempt to extract evidence of special factors through data mining, and to use other data sets to adjust and re-train the model.

Overall, the results of this study demonstrated that lung cancer can be predicted by a person’s disease history. This study tracked a decade of clinical diagnostic records to identify the possible symptoms and comorbidities of lung cancer, to allow for the early prediction of the disease, and to assist more patients by providing an early diagnosis.

Based on the disease diagnostic data that are currently available for this study, the accuracy of the prediction model was close to 86%. In our future study, clinical diagnostic data, blood test data, and physiological data will be integrated and analyzed, which could improve the accuracy of the predictions.

## 6. Limitations

This study used data from Taiwan’s NHIRD and, therefore, the prediction model, may only be applicable in Taiwan. The use of clinical diagnostic data has limitations for clinical decision-making applications; however, it can effectively help early diagnosis and rapid screening. Because the general questionnaire data is not included in the NHIRD, the factors that have been proposed in previous research cannot be used in this model; otherwise, they may have further improved the effectiveness of the model.

## Figures and Tables

**Table 1:** Related work results.

Authors	Algorithm	Number of factors	ROC
Bach et al.	Cox Proportional Hazards Regression	6 age, sex, prior history of asbestos exposure, duration of smoking, average amount smoked per day while smoking, and duration of abstinence from smoking for former smokers	0.72
A. Cassidy et al.	Conditional Logistic Regression	7 Gender, Age, Smoking duration (Years), Family history of lung cancer, Malignancy, Pneumonia, Asbestos exposure	0.71
Spitz et al.	Multivariable Models	6 smoking status, pack-years smoked, age at smoking cessation (former smokers), and number of years since smoking cessation (former smokers)], self-reported physician diagnoses of chronic obstructive pulmonary disease or hay fever, and exposures to asbestos or wood dusts	0.59–0.67
Tammemagi et al.	Cox Proportional Hazards Regression	10 age, level of education, body-mass index (BMI), family history of lung cancer, chronic obstructive pulmonary disease (COPD), chest radiography in the previous 3 years, smoking status (current smoker vs. former smoker), history of cigarette smoking in pack-years, duration of smoking, and quit time (the number of years since the person quit smoking)	0.803
Hoggart et al.	Bayesian Perspective	10 Sex, Education level, Asthma, Family history of cancer, Chr15q25, Chr5p15, Silica, PAH, Metal, Asbestos	0.843
This Study	Deep Neural Network (DNN)	13	0.874

**Table 2:** Subject demographics.

Items		Case Group (with Cancer)		Control Group (No Cancer)		Total
		N = 3316		N = 11,489		
Gender	Male	2220	66.90%	7695	67.00%	9915
	Female	1096	33.10%	3794	33.00%	4890
Age—Group	20–29	5	0.20%	23	0.20%	28
	30–39	59	1.80%	201	1.70%	260
	40–49	222	6.70%	833	7.30%	1055
	50–59	492	14.80%	1672	14.60%	2164
	60–69	753	22.70%	2721	23.70%	3474
	70–79	1198	36.10%	4063	35.40%	5261
	80–89	545	16.40%	1847	16.10%	2392
	90–99	42	1.30%	128	1.10%	170
	100–109	0	0.00%	1	0.00%	1
Total		3316	22.40%	11,489	77.60%	14,805
Pneumonia, organism unspecified ICD-9 = 486	0	2928	88%	10,906	95%	13,834
	1	388	12%	583	5%	971
Respiratory abnormality, unspecified ICD-9 = 786	0	1946	59%	7909	69%	9855
	1	1370	41%	3580	31%	4950
NEC Chronic airway obstruction, not elsewhere classified ICD-9 = 496	0	2742	83%	10,241	89%	12,983
	1	574	17%	1248	11%	1822
Simple chronic bronchitis ICD-9 = 491	0	2413	73%	9104	79%	11,517
	1	903	27%	2385	21%	3288
Acute bronchitis ICD-9 = 466	0	1504	45%	5669	49%	7173
	1	1812	55%	5820	51%	7632
Pulmonary collapse ICD-9 = 518	0	3247	98%	11,393	99%	14,640
	1	69	2%	96	1%	165
Pure hypercholesterolemia ICD-9 = 272	0	2998	90%	9835	86%	12,833
	1	318	10%	1654	14%	1972
Diabetes mellitus without mention of complication, Type II ICD-9 = 250	0	2725	82%	8713	76%	11,438
	1	591	18%	2776	24%	3367
Malignant hypertensive heart disease without congestive heart failure ICD-9 = 402	0	2657	80%	8540	74%	11,197
	1	659	20%	2949	26%	3608
Abscess of lung and mediastinum ICD-9 = 513	0	3306	99%	11,485	99%	14,791
	1	10	1%	4	1%	14
Other unspecified noninfectious gastroenteritis and colitis ICD-9 = 558	0	2395	72%	7570	66%	9965
	1	921	28%	3919	34%	4840
Post inflammatory pulmonary fibrosis	0	3307	99%	11,482	99%	14,789
ICD-9 = 515	1	9	1%	7	1%	16
Acute pulmonary heart disease	0	3306	99%	11,473	99%	14,779
ICD-9 = 415	1	10	1%	16	1%	26

**Table 3:** The coefficient and p value of each factor.

Items	*p* Value	Coefficients	*p* Value
	(Chi Square)	Least Absolute Shrinkage and Selection Operator (LASSO)	Akaike Information Criterion (AIC)
Pneumonia, organism unspecified	1.41 × 10^−34^	0.084021743	0.002287 **
ICD-9 = 486			
Respiratory abnormality, unspecified	4.07 × 10^−28^ ***	0.031545271	0.097719
ICD-9 = 786			
NEC chronic airway obstruction, not elsewhere classified	3.43 × 10^−25^ ***	0.082399648	2.92 × 10^−5^ ***
ICD-9 = 496			
Simple chronic bronchitis	5.28 × 10^−18^ ***	0.040447358	0.006002 **
ICD-9 = 491			
Acute bronchitis	7.3 × 10^−11^ ***	0.033521658	0.110577
ICD-9 = 466			
Pulmonary collapse	4.03 × 10^−7^ ***	0.106741357	0.014834 *
ICD-9 = 518			
Pure hypercholesterolemia	1.56 × 10^−6^ ***	−0.022578026	0.073781
ICD-9 = 272			
Diabetes mellitus without mention of complication, Type II	2.31 × 10^−6^ ***	−0.029885991	9.29 × 10^−5^ ***
ICD-9 = 250			
Malignant hypertensive heart disease without congestive heart failure	8.6 × 10^−6^ ***	−0.043622275	2.35 × 10^−6^ ***
ICD-9 = 402			
Abscess of lung and mediastinum	1.27 × 10^−5^ ***	0.740337834	0.002055 **
ICD-9 = 513			
Other unspecified noninfectious gastroenteritis and colitis	131 × 10^−4^ ***	−0.020047699	0.003216 **
ICD-9 = 558			
Post inflammatory pulmonary fibrosis	5.39 × 10^−4^ ***	0.202481673	0.046431 *
ICD-9 = 515			
Acute pulmonary heart disease	0.016 *	0.272565141	0.006617 **
ICD-9 = 415			

Significant codes: <0.0001 ‘***’, <0.001 ‘**’, <0.01 ‘*’.

**Table 4:** Performance of Different DNN Structures.

Items	AUROC	ACCURACY	SENS	SPEC
1 Layer	0.73	0.7469997	0.58	0.79
2 Layers	0.814	0.7917613	0.62	0.84
3 Layers	0.806	0.7937074	0.63	0.81
4 Layers	0.796	0.8008433	0.58	0.855
5 Layers	0.779	0.788842	0.61	0.854
6 Layers	0.778	0.7502433	0.56	0.88
7 Layers	0.765	0.7538112	0.57	0.89
8 Layers	0.747	0.7129419	0.57	0.88
9 Layers	0.874	0.8541564	0.72	0.86

**Table 5:** Parameters of DNN structure.

Layers	Parameter	Value
1 (Input)	Neuron	13
2–8 (Hidden)	Neuron	2000
	Activation	Relu
	Dropout	50%
9 (Output)	Neuron	2
	Activation	Softmax
Training Times = 1000. Batch size = 100. Optimizer = SGD. Learning rate = 0.1. Momentum = 0.1. Train Type = Feed Forward		

## Data Availability

Data sharing not applicable.
